# Enhanced Transferrin Receptor Expression by Proinflammatory Cytokines in Enterocytes as a Means for Local Delivery of Drugs to Inflamed Gut Mucosa

**DOI:** 10.1371/journal.pone.0024202

**Published:** 2011-09-06

**Authors:** Efrat Harel, Abraham Rubinstein, Aviram Nissan, Elena Khazanov, Mirela Nadler Milbauer, Yechezkel Barenholz, Boaz Tirosh

**Affiliations:** 1 Institute for Drug Research, The Hebrew University of Jerusalem, Jerusalem, Israel; 2 Department of Surgery, Hadassah Hebrew University Medical Center, Mount Scopus, Jerusalem, Israel; 3 The Laboratory of Membrane and Liposome Research, The Hebrew University of Jerusalem, Jerusalem, Israel; Emory Unviersity, United States of America

## Abstract

Therapeutic intervention in inflammatory bowel diseases (IBDs) is often associated with adverse effects related to drug distribution into non-diseased tissues, a situation which attracts a rational design of a targeted treatment confined to the inflamed mucosa. Upon activation of immune cells, transferrin receptor (TfR) expression increases at their surface. Because TfR is expressed in all cell types we hypothesized that its cell surface levels are regulated also in enterocytes. We, therefore, compared TfR expression in healthy and inflamed human colonic mucosa, as well as healthy and inflamed colonic mucosa of the DNBS-induced rat model. TfR expression was elevated in the colonic mucosa of IBD patients in both the basolateral and apical membranes of the enterocytes. Increased TfR expression was also observed in colonocytes of the induced colitis rats. To explore the underlying mechanism CaCo-2 cells were treated with various proinflammatory cytokines, which increased both TfR expression and transferrin cellular uptake in a mechanism that did not involve hyper proliferation. These findings were then exploited for the design of targetable carrier towards inflamed regions of the colon. Anti-TfR antibodies were conjugated to nano-liposomes. As expected, iron-starved Caco-2 cells internalized anti-TfR immunoliposomes better than controls. *Ex vivo* binding studies to inflamed mucosa showed that the anti-TfR immunoliposomes accumulated significantly better in the mucosa of DNBS-induced rats than the accumulation of non-specific immunoliposomes. It is concluded that targeting mucosal inflammation can be accomplished by nano-liposomes decorated with anti-TfR due to inflammation-dependent, apical, elevated expression of the receptor.

## Introduction

Ulcerative colitis and Crohn's disease, collectively termed inflammatory bowel disease (IBD), are the two main chronic relapsing inflammatory diseases of the intestines. While the former is confined to the large bowel, the latter involves the entire gut. The clinical presentation is largely dependent on the disease location and may include diarrhea, abdominal pain, fever, bowel obstruction and bloody stool. Typical complications associated with CD include strictures, abscesses, or fistulas [1], [2]. The etiologies of both ulcerative colitis and Crohn's disease remain unclear and are presumed to result from inherited and environmental factors involving the immune system. Whether the immune system is activated intrinsically (constitutive activation or failure of down-regulatory mechanisms), or by a continuous, mucosal driven stimulation is still unknown [3], [4].

Despite significant progress in IBD therapy, in particular after the introduction of biological drugs such as infliximab [5], [6], patients often suffer disease relapse associated with impairments in their quality of life. This often necessitates a therapy with high doses of anti-inflammatory drugs (steroids or nonsteroidal anti-inflammatory drugs) [7], [8], a regimen commonly accompanied by severe adverse effects. It was postulated by us and others that delivery of potent anti-inflammatory drugs in the closest proximity possible to the inflamed mucosal tissues would improve drug efficacy and reduce toxicity [9], [10], [11]. Still, a major drawback of such colonic delivery systems was their lack of specificity to the inflamed regions within the organ.

It has recently shown that drug enrichment in inflamed mucosa can be accomplished with negatively charged liposomes. Low and high molecular weight antioxidants that were delivered via anionic liposomes to the inflamed mucosa of experimental colitis-induced rats were more effective in attenuating the induced inflammation compared with aqueous solutions of the enzymes [12]. This was probably due to specific attachment of the negatively charged liposomes to the inflamed regions, which led to a prolonged residence time and a better uptake of the antioxidants into the inflamed epithelium. It was also observed that while positively charged liposomes exhibited enhanced adherence to both healthy and normal intestinal mucosa, negatively charged liposomes adhered preferentially to the inflamed (experimental colitis) epithelium of the rat colon [13]. A recent study, conducted in our laboratory, provided evidence that transferrin (Tf) at the luminal region of the inflamed colon, coupled with a typical low luminal pH may be related to the mechanism underlying this preferential adhesion [14]. This observation may also suggest a role for mucosal transferrin receptor (TfR) in IBD. It has already been reported that TfR levels are elevated in activated immune cells, lymphocytes and macrophages [15], [16]. In this study we addressed the question whether TfR levels are subjected to similar regulation in epithelial cells of the colon.

TfR internalizes the serum glycoprotein Tf (80 kDa) by endocytosis [17]. Commonly, TfR is ubiquitously expressed at low levels [18]. Its expression is elevated, however, in rapidly dividing cells, including a variety of human cancers [19], [20]. In addition to its role in iron metabolism it was suggested that TfR may play a role in cellular signaling and proliferation stimuli [21], [22], [23]. We have already shown that the luminal aspect of the inflamed colon epithelium of the rat was enriched with Tf [14], suggesting that the increase in both mucosal Tf levels and TfR expression is associated with the pathophysiology of IBD.

The objectives of the study were to: (***a***) measure the expression levels of TfR in normal mucosa and in IBD; (***b***) elucidate the relationship between typical IBD cytokines and mucosal TfR expression and function in IBD and (***c***) examine the possibility to preferentially target IBD mucosa with immuno-liposomes conjugated with anti-TfR.

## Materials and Methods

Unless stated otherwise, all materials were purchased from Sigma (St. Louis, MO, USA). Solvents were of analytical grade or higher. Water was deionized and ultrafiltered by reverse osmosis (Barnstead Nanopure, Waltham, MA). For detection of human and rat TfR we used the monoclonal anti-TfR (H68.4, Abcam, Cambridge, MA). Goat anti-mouse IgG Alexa Fluor® 647 was purchased from Invitrogen (Carlsbad, CA, USA). For detection of human E-cadherin we used Mouse anti human E-cadherin BD Transduction Laboratories (NJ USA). Rabbit anti-GRP78 (BiP) antibody was obtained from Abcam (Cambridge, MA, USA). Horseradish peroxidase (HRP) - conjugated secondary antibodies were purchased from Jackson ImmunoResearch Laboratories (Suffolk, UK) and Santa Cruz Biotechnology (Santa Cruz, CA, USA). Hydrogenated soybean phosphatidylcholine (HSPC), phosphatidylglycerol (HSPG) and 1, 2-Dioleyl-sn-glycerol-3-phosphoethanolamine- N-carboxyfluorescein (PE-CF) were purchased from Avanti Polar Lipids (Alabaster, AL, USA). NHS-PEG-DSPE [3-(N-succinimidyloxyglutaryl) aminopropyl, polyethyleneglycol-carbamyl distearoylphosphatidyl-ethanolamine] was obtained from NOF Corporation (Tokyo, Japan). Elisa kit for rat TNFα was obtained from R&D Systems, Inc. (MN, USA) and the mouse Cytokine Screen ELISA from Quansys Biosciences (UT, USA).

### Ethics statement

Inflamed and non-inflamed human colons were obtained as slides of paraffin embedded from the Department of Surgery, Hadassah - Hebrew University Medical Center, Mount Scopus. This part of the study was approved by the Independent Ethical Committee (IEC, Helsinki Committee)-Protocol # HMO-0512-08. Adult patients operated on for inflammatory bowel disease were offered participation in the trial. Eligible patients signed an IEC-approved consent form.

All animal studies were conducted in accord with the Principles of Laboratory Animal Care (NIH publication #85-23, revised1985) under protocol no. MD-10603-4. The joint ethics committee (IACUC) of the Hebrew University and Hadassah Medical Center approved the study protocol for animal welfare. The Hebrew University Animal Facility is an AAALAC international accredited institute (#1285).

### Rats, maintenance and euthanasia

Male Sabra rats (200–225 g), obtained from Harlan Laboratories, Ein Kerem (Jerusalem, Israel) breeding farm, were kept under constant environmental conditions (22°C, 12 h light/dark cycles) and fed with standard laboratory chow and tap water. Anesthesia was performed by an intraperitoneal injection of a mixture of 100 mg/kg body weight of ketamine (Ketaset, 0.1 g/mL, Fort Dodge, IA, USA) and xylazine HCl (20 mg/mL). Euthanasia of anesthetized rats was carried out by chest wall puncturing.

### Induction of experimental colitis in the rat and inflammation severity assessment

Twenty-four hours prior to colitis induction, the rats were deprived of food, but allowed free access to water. Experimental colitis was induced, under light anesthesia (isoflurane inhalation), by a rectal slow (20 seconds) instillation of 30 mg of dinitrobenzensulfonic acid (DNBS) dissolved in 1 mL of 50% v/v ethanol through a flexible, perforated foley catheter (0.3 mm outer diameter). Immediately after the hapten administration the catheter was removed and the rats were left in an inverted position for additional 40 seconds. Four days after colitis induction (maximal onset of inflammation as identified in preliminary studies) the rats were anesthetized as above, their abdomen was cut open and a 0.5 ml blood was withdrawn from the portal vein for hematological analysis (RBC, hematocrit and hemoglobin analyses). The colons were removed and rinsed with normal saline. Inflammation severity was scored (wet weight, dimensions, edema, hyperemia ulceration area and stool viscosity prior to the saline rinse) [12], [24]. The control (healthy) group was treated similarly with a normal saline solution replacing the DNBS ethanolic solution. Homogenates of colon tissues obtained from of inflamed or non-inflamed regions one or four days after colitis induction were analyzed for TNFα by commercial ELISA kit (Eli-pair, Diaclone, France).

### TfR immunofluorescence analysis in rat colon mucosal specimens and in human biopsies

Mucosal specimens from isolated rat colons (healthy or inflamed regions) were rinsed with a 4% w/v aqueous sucrose solution and fixated with 12% v/v formaldehyde in PBS. After a PBS rinse the fixated tissues were preserved in a 20% w/v sucrose solution until sectioning. Prior to sectioning the specimens were dehydrated (ethanol and PBS). Sectioning (10–12 µm) was performed by embedding in paraffin blocks and press-heating in 40 nM Tris/1 Mm EDTA buffer pH 9, at 125°C for 2.45 minutes, followed by 27 min at 90°C. Nonspecific staining was blocked by 2% BSA in PBS. Immunostaining of the fixed colonic specimens from the rat or human biopsies was performed by their incubation (overnight, 4°C) with diluted (1∶500) mouse anti-human TfR followed by incubation (1 h, room temperature) with diluted (1∶800) goat anti-mouse IgG- Alexa 647. Nuclei were stained with 0.1 mg/ml of Hoechst dye. Fluorescence was monitored by confocal microscopy (Carl Zeiss, Germany).

### Western analysis of TfR expression in the colonic mucosa of the rat

Mucosal specimens from isolated rat colons from either DNBS-induced or saline-treated rats were scraped with glass slides, collected in a microcentrifuge test-tube containing a lysis buffer (RIPA), shaken vigorously (15 min, 4°C) and centrifuged (10 min, 14,000 rpm, 4°C). The clear supernatant was collected and its protein content was determination (Bradford). A volume equivalent to 15 µg of protein was boiled in reduced Laemmli sample buffer, analyzed by 12% SDS-PAGE and electrotransferred to a nitrocellulose membrane. Membranes were blocked with 5% non-fat milk in PBS containing 0.05% Tween 20 (PBST) for 1 hour at room temperature and stained with mouse anti human TfR. HRP-conjugated goat anti mouse was used as a secondary antibody. Rabbit anti BiP was used as loading control, followed by HRP-conjugated goat anti rabbit. Proteins were visualized by chemical luminescence (EZ-ECL, Biological Industries, Israel).

### Preparation of cytokine rich medium (CRM)

Male C57BL/6 mice (20 g) or male Sabra rats (250 g) were sacrificed, their spleen was removed immediately into 10 ml of PBS (RT) pounded and sieved through a cell strainer. The sieved homogenate was centrifuged (5 min, 1,250 rpm) and red blood cells were removed by incubation in lysis buffer (20 ml, 5 min, RT). After centrifugation the supernatant was discarded and the cells-containing pellet was resuspended immediately in 10 ml of warm DMEM (no serum). 10×10^6^ cells/ml were plated in the presence of 100 µg/µl of lipopolysaccharide (LPS) in a 96 wells plate. After incubation (24 h, 37°C, 5% CO_2_) the content of each well was aspirated, centrifuged (4,000 rpm, 20 min, 4°C), the supernatants collected, and stored at −80°C until use for inflammation induction. The cytokine content of the CRM was characterized by multi cytokines elisa (QUANSYS) according to the manufacturer's protocol. CRM was positive for IL-1α, IL-1β, IL-3, IL-6, IL-10, Interferonγ, TNFα, MIP-1 α, RANTES and negative for IL-2, IL-4, IL-5, IL-12p70, IL-17, MCP-1, GM-CSF.

### Preparation and characterization of negatively charged fluorescently-tagged PEGylated immunoliposomes

Seven different liposomal formulations were prepared by the ethanol injection method [14]. Lipid composition is specified in **Table 1**. The formulations differed from each other by the amount of antibodies (Ab) on their surface. All liposomes were tagged fluorescently with 0.5 mole% of 18∶1, 1,2-dioleoyl-sn-glycero-3-phosphoethanolamine-*N*-(carboxyfluorescein). After dialysis against PBS, the liposomes were extruded stepwise with a final extrusion through 100 nm pore polycarbonate filter and kept in PBS at 4°C until use. The Ab was either mouse anti human TfR (anti-TfR) or goat anti-mouse IgG (Anti IgG, served as non-specific controls). The Ab was conjugated to the liposomes, immediately after their preparation, with the aid of 3-(N-succinimidyloxyglutaryl) aminopropyl, polyethyleneglycol-carbamyl distearoylphosphatidyl-ethanolamine (NHS-PEG-DSPE) spacers, which was added to the lipids before hydration preincorporated into the liposomal formulation, at a ratio of 5 Mol% (**Scheme S1**). The coupling reaction was terminated with 100 mM of TRIS buffer.

**Table 1:** Materials used for the various liposomal formulations.

Liposomal Formulation Name →	No Ab		Anti TfR 50		Anti TfR 5		Anti TfR 1		Anti IgG 100		Anti IgG 10		Anti IgG 1	
Composition	Mol%	mM	Mol%	mM	Mol%	mM	Mol%	mM	Mol%	mM	Mol%	mM	Mol%	mM
HSPC	64.5	12.9	64.5	12.9	64.5	12.9	64.5	12.9	64.5	12.9	64.5	12.9	64.5	12.9
HSPG	30	6	30	6	30	6	30	6	30	6	30	6	30	6
NHS- PEG-DSPE	5	1	5	1	5	1	5	1	5	1	5	1	5	1
FC-PE	0.5	0.1	0.5	0.1	0.5	0.1	0.5	0.1	0.5	0.1	0.5	0.1	0.5	0.1
Ab amount (µg) -theoretical	-		50 µg		5 µg		1 µg		100 µg		10 µg		1 µg	

**Abbreviations:**

**CRM**: cytokine rich medium.

**HSPC**: Hydrogenated soy phosphatidylcholine.

**HSPG**: Hydrogenated soy phosphoglycerol (HSPG).

**NHS-PEG-DSPE**: 3-(N-succinimidyloxyglutaryl) aminopropyl, polyethyleneglycol-carbamyl distearoylphosphatidyl-ethanolamine.

**FC-PE**: 18∶1, 1,2-dioleoyl-sn-glycero-3-phosphoethanolamine-N-(carboxyfluorescein).

**Figure 1:**
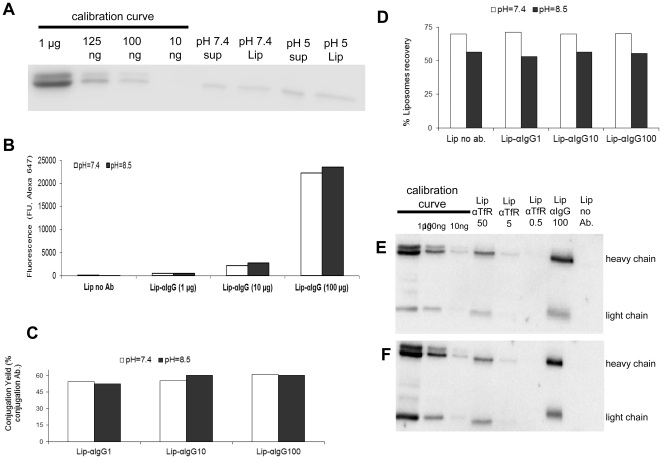
Optimization of anti-TfR conjugation to the liposomes. (A) Identifying the optimal pH (5 or 7.4) for the imunoliposomal preparation by Western blot analysis; (B) Fluorescence measurements of Alexa 647 (anti IgG) at different concentrations (1, 10,100 µg) conjugated to liposomes in pH = 7.4 or pH = 8.5; (C) Fluorescence assessments of the conjugation yield of the anti IgG antibodies to the liposomes at pH = 7.4 or pH = 8.5; (D) Recovery of liposomes after ultracentrifugation following antibody conjugation reaction at pH = 7.4 or pH = 8.5; (E, F) Western blot analysis for anti-TfR or anti-IgG conjugated antibodies (E, upper blot) and antibody content in the supernatant (F, lower blot) after ultracentrifugation (pH = 7.4).

Since the functional group for attaching the antibody (NHS coupled to PEG-DSPE) is prone to hydrolysis prior to antibody conjugation, we evaluated two pH conditions acidic (pH = 5) and neutral (pH = 7.4) for liposome preparation. After liposome extrusion, the antibody was coupled at pH value same as for liposome fabrication (overnight, room temperature). Following quenching of non reacting NHS groups with TRIS, non-bound Ab was removed by ultracentrifugation (55,000 RPM, 2 h). Ultracentrifugation did not affect liposome size (data not shown). The pellet containing the immunoliposomes was resuspended in PBS and kept at 4°C until use. After resuspension of the liposomal pellet, an aliquot was removed and immediately boiled in reduced Laemmli sample buffer. An equivalent portion of the supernatant, which contains unconjugated Ab as well as non-precipitated immunoliposomes, was also boiled in reduced Laemmli sample buffer. Western blotting analysis for the presence of antibody heavy and light chains indicated similar efficiency of antibody coupling at either pH = 7.4 or pH = 5.0. We estimate the recovery of the immunoliposomes by precipitation at approximately 50%, as the antibody amount in the pellet (labeled as Pellet) or the supernatant (labeled as Sup) was similar (**figure 1A**).

To further evaluate the effect of pH on the conjugation reaction, we incorporated into the liposomes a carboxyfluorescein-tagged lipid and used Alexa 647-tagged Ab. This double staining with orthogonal fluorophores allowed a single step quantitation of the conjugated Ab and the liposomes. Increasing amounts (1, 10 or 100 µg) of Alexa 647-labeled antibodies were incubated with the carboxyfluorescein-tagged liposomes at pH = 7.4 or pH = 8.5. Unbound Ab was removed by ultracentrifugation and the fluorescence of either of the two fluorophores was determined in the pellet and the supernatant. The Alexa 647 concentration-dependent increase in the pellet's fluorescence at λ = 647 nm, at two pH values is shown in **Figure 1B**, indication on an Ab increase in the pellet. Again, we observed a yield of ∼50% regardless the antibody amount or the pH conditions (**Figure 1C**). However, the efficiency of ultracentrifugation was better at pH 7.4 than 8.5 (70% and 55%, respectively) (**Figure 1D**). Therefore all liposomal preparations were conducted at pH = 7.4. Similar results were obtained for the mouse anti human TfR (**Figures 1E and 1F**). Based on the ultracentrifugation recovery values, the conjugation efficacy was estimated to be approximately 100%, as the amounts of Ab in both liposomal pellet and supernatant were similar to the amounts of the liposomes themselves in the corresponding fractions. This indicated that the vast majority of Ab molecules in the supernatants were conjugated to liposomes that failed to precipitate. Thus, the ultracentrifugation step was the main limitation in the preparation of the immunoliposomes.

### Physical characterization of the immunoliposomes

Size distribution and zeta potential were analyzed after dilution (1∶100) with sodium nitrite 0.15 M by Zetasizer (3000 HS, Nano-ZS, Malvern, UK). The size and zeta potential of the seven formulations (**Table 1**) were similar, with an average size of 105.6±7 nm and a zeta potential of −14.1±3.3 mV. Fluorescence level of the liposome was measured (Synergy HT, BioTek, VI, USA) at λex = 485 nm and λem = 528 nm. Liposomes phospholipids content was measured by determining their phospholipid phosphorus concentration as described elsewhere [25].

### Liposomal antibody load quantification by Western blot

Twenty µl of the immunoliposomes were boiled in a reduced Laemmli sample buffer and loaded onto 12% SDS-PAGE resolution gel. Following electrotransfer to a nitrocellulose membrane, Ab heavy and light chains were detected by HRP-conjugated antibodies. Their amounts were assessed with the aid of a 4-points calibration curve, prepared in a similar manner, using known amounts of purified Ab.

### Cell proliferation and TfR levels analysis by flow cytometry

Low passage Caco-2 cells were purchased from the ATCC (Manassas, VA). Cell proliferation was measured by carboxyfluorescein diacetate succinilimide ester (CFSE, Invitrogen, Merelbeke, Belgium) dilution assay. In this assay, as the cell proliferates the entrapped CFSE is diluted. Hence, proliferation is indicated by low CFSE levels. Caco-2 cells, harvested by 2 mM of EDTA in PBS, were resuspended in PBS containing 1% BSA and incubated (7 min, 37°C) with 10 µm of CFSE, after which the cells were washed and resuspended in a culture medium. The cells were cultured for 24 h and the medium was replaced by a fresh one containing the following recombinant human cytokines TNFα, IL1β and IL-6 (Peprotech, Israel), separately, mixed together or CRM and incubated for additional 24 h. Because CRM contained LPS, LPS was added to the incubation medium of the control studies involving Caco-2. TfR was stained with mouse anti-human TfR following by Alexa 647 labeled goat anti-mouse IgG. TfR expression and proliferation analysis was performed by flow cytometry (LSR II, Becton, Dickinson Biosciences, ON, Canada).

### Tf uptake by iron-starved Caco-2 cells

Low passage Caco-2 cells were cultured in DMEM containing 10% fetal bovine serum (FBS), 100 IU/ml of penicillin, 1% L-glutamine, 1 mM sodium pyruvate and 1% non essential amino acids. Cells were plated on μ- slide 8 wells (Ibdi, Germany) at a concentration of 3×10^4^ cells/well. After the addition of CRM the plates were incubated for 24 h at 37°C. Fifty µg of Alexa 594-labeled Tf (Tf-594, Invitrogen, CA, USA) was added and plates incubated for additional 24 h. The cells were then rinsed twice with PBS and Tf uptake was visualized by confocal microscopy (Zeiss, Germany). Iron starvation was accomplished by the addition of 10 µM Desferal® to the incubation medium. Non-starved cells served in the control studies.

### Uptake of the anti TfR- immunoliposomes by Caco-2 cells- confocal microscopy analysis

Iron-starved Caco-2 cells were cultured on μ-slide 8 wells plates (Ibdi, Germany) and incubated in the presence or absence of proinflammatory cytokines (24 h, 37°C), followed by a 4 h incubation (37°C) with 20 or100 µl of anti-TfR- immunoliposomes. After incubation the cells were washed twice with PBS and imaged by confocal microscopy (Zeiss, Germany). Immunoliposomes carrying anti IgG antibodies served as a negative control.

### Flow cytometry analyses for the uptake of Anti-TfR liposomes by Caco-2 cells

CaCo-2 cells, pretreated with CRM and desferal for 24 hours were incubation for 6 h with naked (no Ab.) liposomes, Lip-αIgG100, lip-αTfR50, all fluorescently labeled formulations were of equivalent florescence level. Flow cytometry analysis was performed on LSR II instrument (Becton-Dickinson).

### Uptake of the anti TfR- immunoliposomes by colon mucosa

The colons specimens of either anesthetized DNBS-induced or healthy rats were removed and rinsed with normal saline and incubate (30 min, 37°C) with 2 mM of dithiothreitol to remove the mucous lining which covers the epithelium. Everted sacs (1 cm) were prepared and incubated (3 h, 37°C, while shaking) in 100 µl of the immunoliposomes, or liposomes without Ab (**Table 1**), suspended in 1 ml of PBS. The sacs were then washed twice with PBS and homogenized, on ice in 1 mL of acidic isopropanol. The homogenates were centrifuged (25,000 g, 30 min, 4°C), and the clear supernatant was collected. After the addition of a borate buffer (pH = 9), fluorescence was measured (excitation: 485 nm, emission:521 nm) and liposomes concentrations were calculated with the aid of a 5 points calibration curve which was made of increasing amounts of the liposomes in acidic isopropanol.

## Results

### TfR expression was elevated in human specimens from IBD patients and in the colonic epithelium of rats induced with experimental colitis

**Figure 2:**
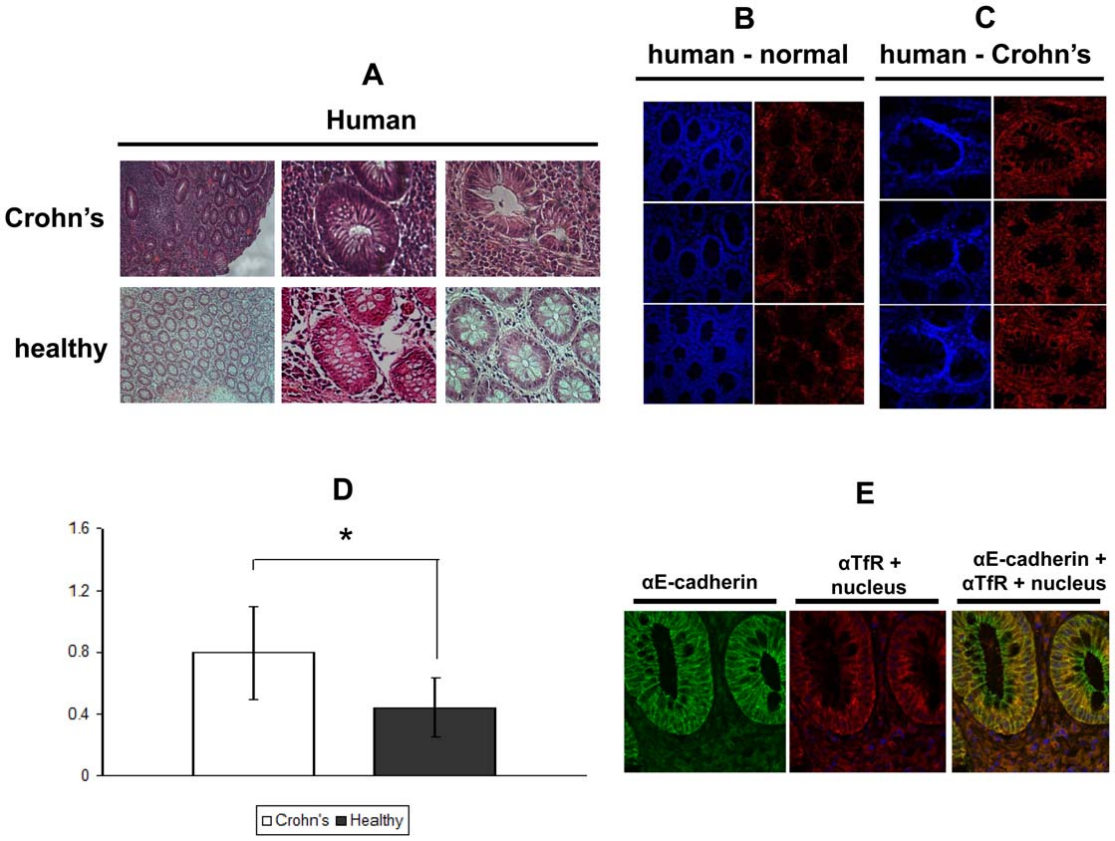
Enhanced expression of TfR in inflamed colonic epithelium in humans. (A) Typical hematoxylin and eosin staining of human biopsies taken from Crohn's disease patients and patients with non-inflamed colonic mucosa.TfR immunostaining of transverse sections from human biopsies taken from non-inflamed colonic mucosa (B) and from lesions of Crohn's disease patients (C). Left panels (of B and C): nucleus staining (blue). Right panels (of B and C): TfR staining (red). Magnification: ×25; (D) Quantification of the fluorescence intensity (Image Pro Plus) in the specimens taken from the Crohn's patients (N = 9) and non-inflamed (“normal”) patients (N = 12) is shown on the left; (E) Anti-E-cadherin (left panel, green) staining of inflamed tissue, staining of TfR (red) and nuclei (blue) (middle panel), merge (right panel).

**Figure 3:**
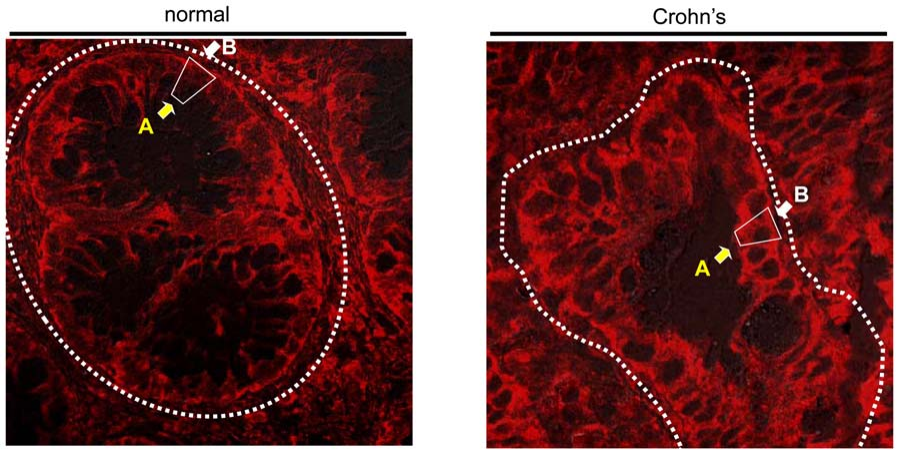
TfR is expressed at the apical aspect of the inflamed colonic epithelium in humans. Larger magnification (×63) of (Figure 2B) immunostained for TfR. Left: a transverse section from non-inflamed colonic mucosa. Right: a transverse section from a Crohn's disease patients, the basolateral membrane of the colon crypts is marked with white arrows, while the apical membrane is marked with yellow arrows.

To examine the relationship between epithelial inflammation in the colon and TfR expression, we analyzed human colon biopsies from Crohn's disease patients. Specimens were taken from active inflamed areas and compared to histological normal colonic mucosa form patients with diseases other than IBD. Inflammation was verified histologically by standard H&E staining (**Figure 2A**). In 7 of 9 IBD patients elevated signal for TfR was obtained compared to normal tissues (a total of 12 samples) (**Figure 2B, 2C and 2D**). This signal may originate from the epithelial lining as well as from the infiltrated immune cells or other cell types in the specimen. Costaining with the specific epithelial marker E-cadherin demonstrated that the vast majority of the TfR signal emanates from the epithelial lining (**Figure 2E**), suggesting an increased expression of TfR in the epithelial cells under conditions of inflammation. Interestingly, while in the normal colonic mucosa TfR expression was observed at the basolateral surface, in the biopsies taken from active IBD patients TfR was clearly confined also to the apical aspect of the mucosa (**Figure 3**).

**Figure 4:**
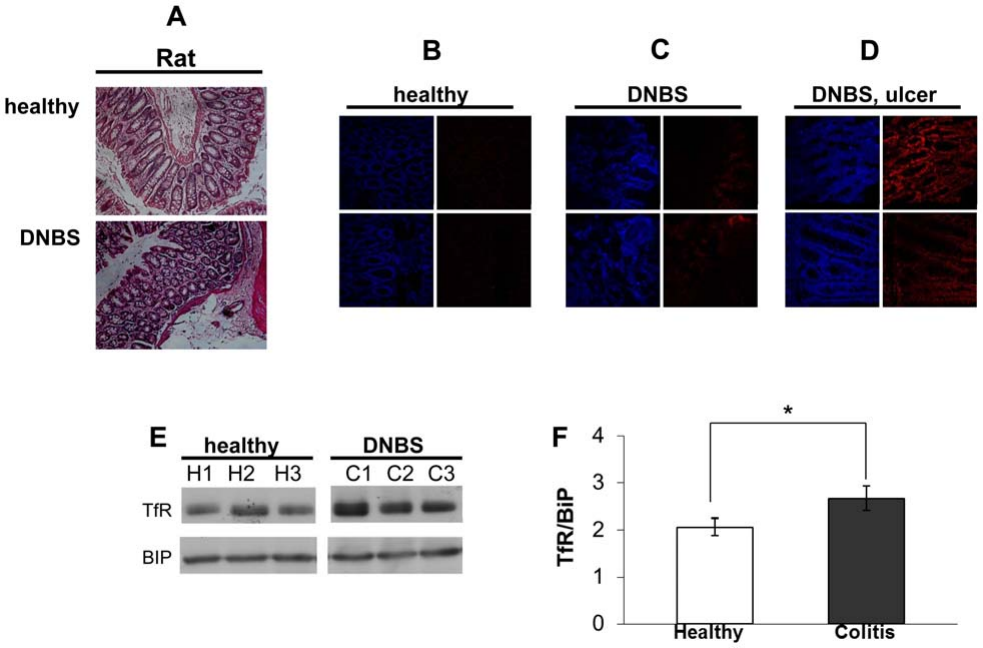
TfR expression is elevated in the mucosa of DNBS induced rats. (A) Hematoxylin and eosin staining of rat colon specimens taken from DNBS-induced rats 4 days after colitis induction and from healthy rat colonic mucosa.TfR immunostaining of transverse sections taken from the mucosa of vehicle treated (B), DNBS-induced (C) and ulcerated region in the DNBS-induced (D) rats. Left panels: nucleus staining (blue). Right panels: TfR staining (red). Magnification: ×25; (E) immunoblot analysis of protein extracts (40 µg per lane) taken from the colon mucosa of sham treated (“healthy”) or inflamed mucosa from DNBS-induced rats. Anti-TfR (95 kDa) was used for staining. (F) Blots quantification was performed by densitometry and normalized to BiP (78 kDa). *P-value* <0.05 was obtained by Mann-Whitney test.

To further examine whether TfR expression is affected by inflammation conditions we established a DNBS-induced colitis in rats. TfR expression was also measured in the colonic epithelium of rats 4 days after experimental colitis induction (a period of time which was required for the mucosa to recover from the local damage caused by the ethanolic vehicle of the control group). Inflammation was verified by H&E staining (**Figure 4A**) and further characterized directly by measuring colon mucosa edema ulceration and change in colon weight and length, and indirectly by monitoring diarrhea, rat weight and hyperemia (Figure S1, A). An elevation in mucosal TfR expression accompanied the DNBS induced inflammation, compared with the sham treatment with highest levels in the ulcerated regions of the mucosa (**Figure 4B** **,** **3C and 3D**). This observation was verified by a Western blot of total protein extraction from the inflamed and vehicle-treated rat mucosa. As seen in **Figure 4** **(panels E and F)**, higher TfR levels were observed in the former.

The elevated mucosal TfR level in the inflamed colons was expected due to the inflammation-driven bleeding. This theoretically should lead to the dwindling of iron depots, which signal via RNA-binding iron regulatory proteins (IRP) signaling to increase TfR translation [26], [27]. To account for this possibility blood samples of 3 vehicle-treated and 4 colitis-induced rats were collected, sera was separated and tested, hematologically, for anemia. Surprisingly, no difference between the two groups could be observed for both red blood cells count and hemoglobin and hematocrit levels (see **Figures S1 and S2**), suggesting a direct impairment involved in the mucosal TfR elevated expression.

### Proinflammatory cytokines promotes TfR expression in Caco-2 cells

**Figure 5:**
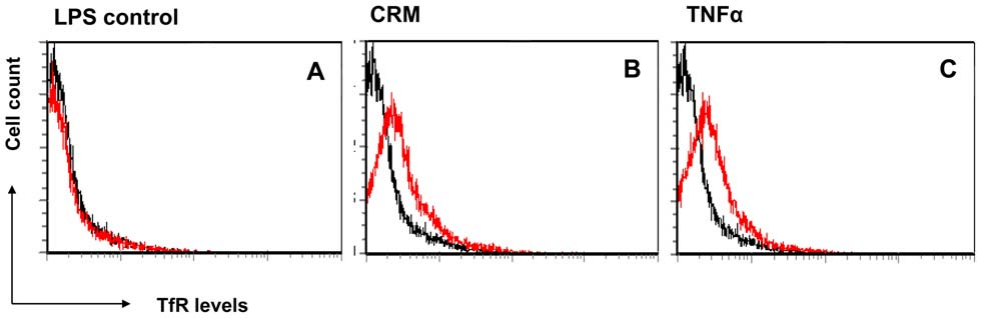
Proinflammatory cytokines increase TfR levels in Caco-2 cells. Flow cytometry analysis of Caco-2 cells following 48 h incubation with LPS (negative control, A), CRM (B), or TNFα (C).

To better understand the involvement of induced inflammation on TfR expression, we decided to explore, at the cellular level, the role of proinflammatory mediators in TfR expression. The human colon carcinoma Caco-2 cell line is a widely used model for colon epithelium. Flow cytometry analysis of the Caco-2 cells incubated with CRM revealed elevated cell surface TfR levels (**Figure 5A, 5B**). It has been shown that the exposure of Caco-2 cells to TNFα (a proinflammatory cytokine, playing a major role in the pathogenesis of IBD) increased iron ions uptake from the incubation medium [28]. We therefore speculated that TNFα was responsible, at least in part, for the CRM-induced increase in TfR expression. Indeed incubation of the Caco-2 cells with recombinant TNFα resulted in a similar increase in TfR expression (**Figure 5C**). We conclude that proinflammatory cytokines promote the cell surface expression of TfR in colon epithelium.

**Figure 6:**
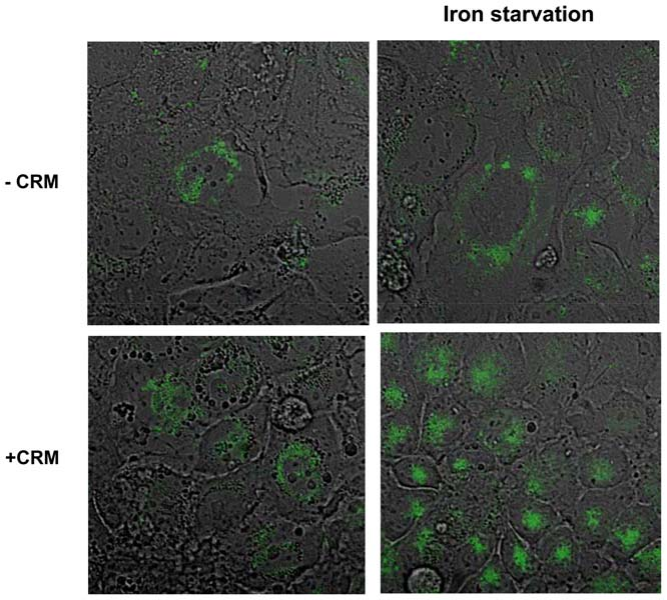
Proinflammatory cytokines promote Tf uptake by CaCo-2 cells. The effect of CRM (lower panel) and iron starvation (100 µM Desferal, right panel) on tagged (Alexa 594) Tf uptake by Caco-2 cells as imaged by confocal microscopy (×25).

Higher levels of TfR are not necessary associated with Tf uptake enhancement. For that purpose we imaged by confocal microscopy the uptake of fluorescent Tf by Caco-2 cells. **Figure 6** shows an enhanced Tf uptake caused by the presence of the CRM cocktail, an effect which was even augmented when the cells were deprived of iron by the addition of 100 µM of Desferal to the incubation medium. Similar observation was obtained when the study was repeated with IEC-6 cells (resemble normal, intestinal epithelium, data not shown). These observations support the assumption that iron uptake in the colon epithelium is affected by local inflammation due to the increase in cell surface TfR.

### Hyper proliferation was not involved in the cell-surface TfR expression

**Figure 7:**
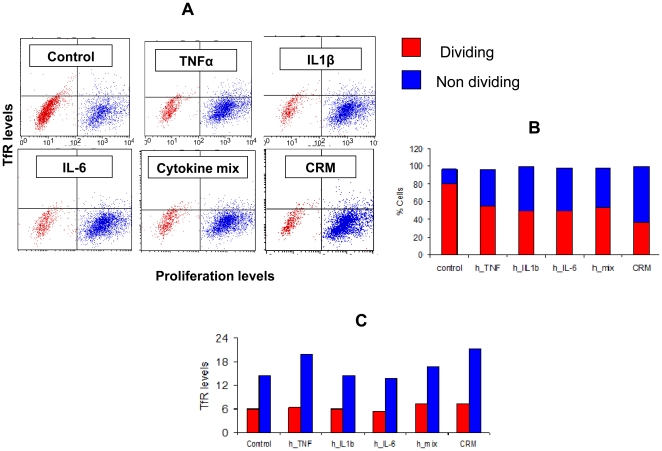
Increased TfR expression in CaCo-2 cells is not due to enhanced proliferation. Flow cytometry analysis of CaCo-2 cells double stained for CFSE dilution assay and TfR (mouse anti-human TfR stain), after incubation with the following inflammation mediators: TNFα, IL1β, IL-6, a mixture of the three, or CRM from mouse source. (A) Dot plot of stained TfR levels and the proliferation marker, CFSE. Dividing cells are marked in red, non-dividing cells are marked in blue; (B) Quantification of the dot plot shown in A; (C) TfR quantification in the dividing (red) and non-dividing (blue) cell populations.

In immune cells activation often triggers proliferation, which necessitates higher uptake of iron. Therefore, we tested whether cytokine-driven enhanced TfR expression was a consequence of hyper proliferation, rather than a direct effect on TfR synthesis. To address this, we monitored Caco-2 cell proliferation by the carboxyfluorescein succinimidyl ester (CFSE) dilution assay. A double staining of the cells in the presence or absence of human TNFα, IL1β, IL-6, mix thereof or CRM, revealed a reduction in the fraction of proliferating cells (**Figure 7**). Interestingly, TfR levels were higher in the non-proliferating cells (CFSE high), compared with the CFSE low population, indicating that the proinflammatory cytokines had a net effect on the elevation in cellular TfR expression, which was not related to their proliferation.

### Cellular uptake of immunoliposomes is increased compared with naked liposomes with and without cytokine

**Figure 8:**
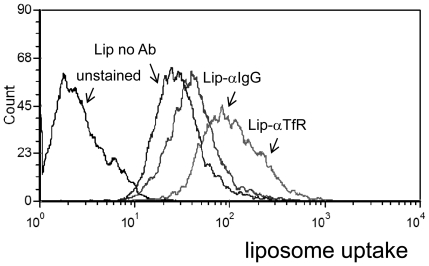
Anti-TfR antibodies maintain functionality when conjugated to the liposomes. Flow cytometry analysis of CaCo-2 cells pretreated with CRM and desferal for 24 hours following 6 h incubation with fluorescently-labeled negative liposomes.

**Figure 9:**
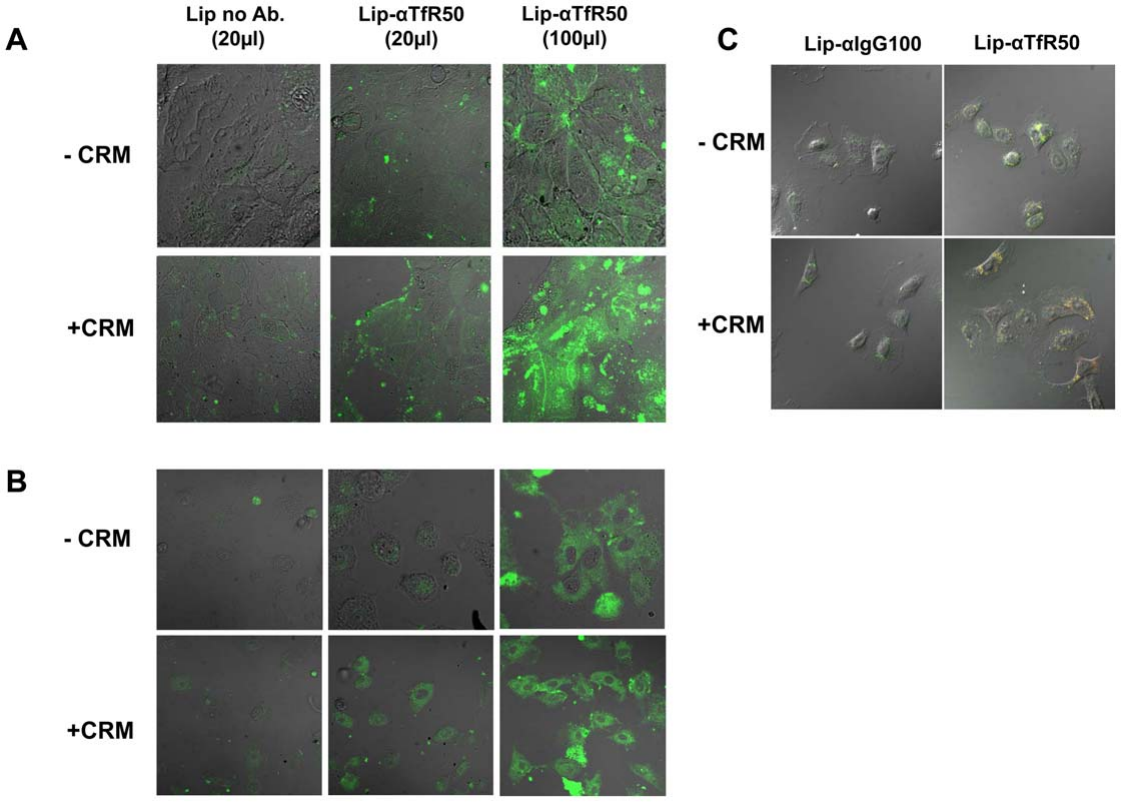
Cytokine treated CaCo-2 cells uptake better the immunoliposomes than naked liposomes. Representative images of confocal scan microscopy of fluorescently-tagged immunoliposomes conjugated to anti-TfR Ab (center and right panels) or naked (no Ab) negative liposomes (left panel) after 24 h of incubation with non stimulated (upper panels) or pre-incubated (lower panels) with CRM. Caco-2 cells are in panel A; IEC-6 cell are in panel B. Epifluorescence images following 10 h incubation of Lip-αIgG or Lip-αTfR in the presence or absence of CRM (C).

Flow cytometry was also used for comparing the ability of the immunoliposomes to direct themselves to CaCo-2 and IEC-6 cells compared to the uptake of immunoliposomes bearing a nonspecific Ab and with that of naked (no Ab) negatively charged liposomes. Caco-2 cells were incubated with the various preparations at equivalent fluorescence level. **Figure 8** shows that anti-TfR immunoliposomes accumulated to a higher level in Caco-2 cells. Since the interaction between membrane TfR and anti-TfR (either free or conjugated to the liposomal vehicle) should induce endocytosis, it was expected that targeting the cellular TfR would facilitate not only liposomal adherence, but also provoke their intracellular uptake. To demonstrate this directly we incubated either fluorescently-labeled immunoliposomes or naked liposomes with Caco-2 or IEC-6 cells at equivalent fluorescence level. The incubation was conducted in the presence or absence of CRM. Liposome uptake was assessed by confocal microscopy. When uptake was compared, the anti-TfR liposomes entered the endocytic pathway to higher efficiency than of the naked liposomes (**Figure 9A and B** **, left and middle panels**). Increase of the amount of liposomes was resulted in increased uptake, indicating that the experiment was not performed under saturation conditions (**Figure 9A and B** **, right panel**). Anti-TfR liposomes were also found to be superior in their intracellular uptake when compared to liposomes equipped with non-specific antibody (**Figure 9C**).

**Figure 10:**
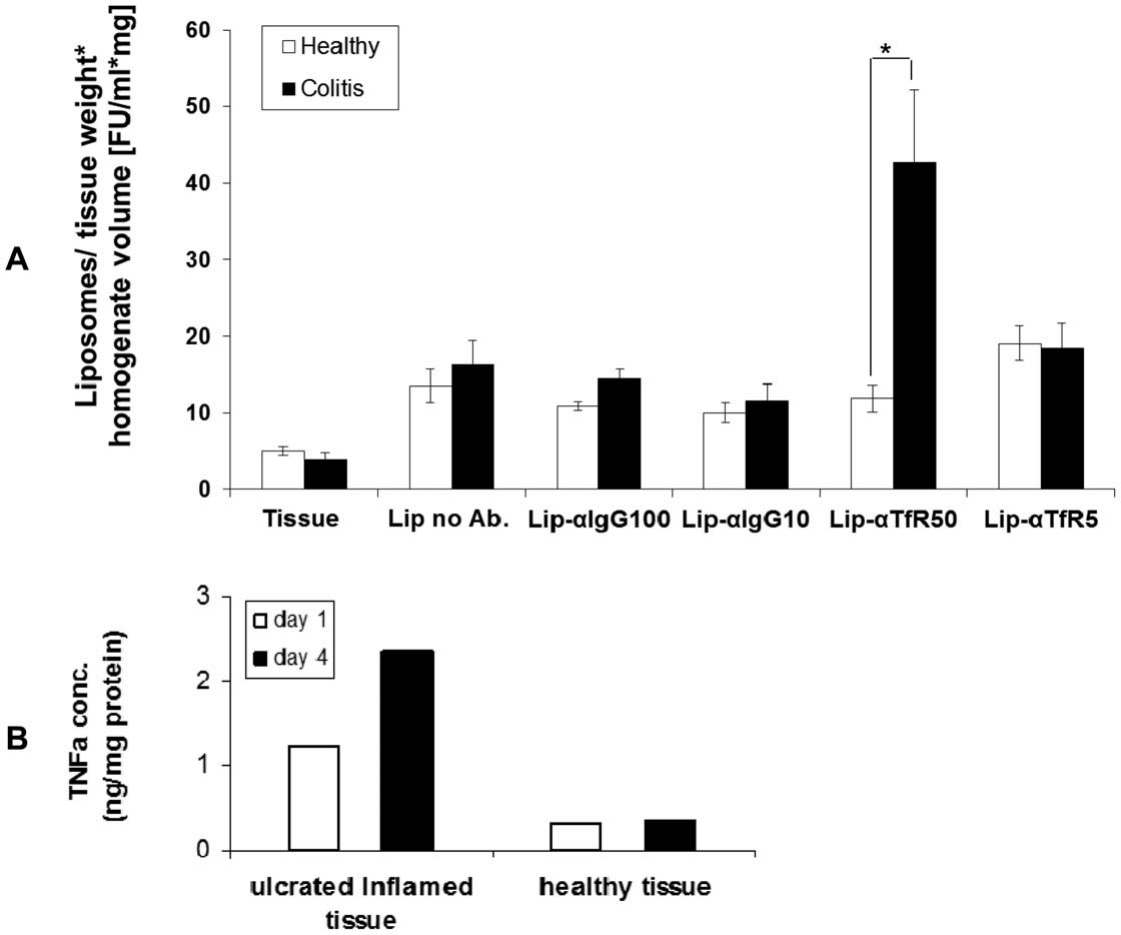
Anti-TfR immunoliposomes adhere preferentially to inflamed mucosa. (A) Ex vivo adherence of two formulation bearing anti-TfR antibody (“Lip-αTfR5 “ and “lip-αTfR50” respectively), two negative control formulations, bearing non-specific antibody (“Lip-αIgG10” and “Lip-αIgG100” respectively) and naked liposomes (no antibody, “lip no Ab”) to everted gut-sacs taken from DNBS-treated or vehicle treated rats. Shown are the averages of 3 independent experiments ± S.E.M. * P-value<0.05 was obtained by t- test. (B) TNFα levels analyzed by ELISA of inflamed and non-inflamed homogenate tissue obtained from experimental induced colitis one or four days after colitis induction.

Lastly, the targeting capability of the novel immunoliposomes was tested in everted gut-sac preparations, taken from inflamed colon of the DNBS induced rat and from vehicle treated control rats. For that purpose 5 formulations were prepared: two formulation of immunoliposomes bearing 5 or 50 µg anti-TfR antibody, two negative control formulations, bearing 10 or 100 µg non-specific antibody and naked liposomes (no antibody). We observed that the anti-TfR immunoliposomes formulation adhered significantly better (up to 4.5-fold) to the inflamed colon epithelium compared with the three non specific formulations (**Figure 10A**). This was in good agreement with the local TNFα concentration in the mucosa (**Figure 10B**).We conclude that immunoliposomes directed to the TfR may provide a means for apical targeting to inflamed colon mucosa.

## Discussion

In recent years, major improvements were achieved in the management of IBD. The incorporation of immunosuppressive agents as well as anti-TNFα antibodies improved IBD outcomes and quality of life. However, toxicity and treatment failure resulting in surgery are still major issues in the management of IBD. Ferrying drugs directly to the colon (“colonic delivery systems”) lacks the ability to target the inflamed mucosa and new approaches that will allow mucosal accumulation of antiinflammatory drugs after oral or rectal administration are sought after [9], [29]. Employing a proteomic approach, in the search for suitable mucosal targets, we identified recently that Tf accumulates at the apical side of the inflamed gut mucosa [14]. This observation impelled us to investigate whether Tf plays a physiological or pathological role in regulating iron metabolism during colonic inflammation. An obvious notion was to explore a possible linkage between inflammation and local TfR expression. Moreover, because TfR levels are elevated along with the local accumulation of immune cells [16], we addressed the question whether similar TfR increase, with potential medical merit, is observed in epithelial cells, in addition to its systemic elevation.

TfR is encoded by two genes producing two types of the receptor - TfR1 and TfR2. The affinity of the former to Tf is 25-fold higher that the affinity of the latter. At the transcriptional level TfR1 expression is controlled by the intracellular level of iron. When the levels of iron drop, RNA- binding iron regulatory proteins (IRPs) become active and bind to an IRE element in the mRNA of TfR1. This IRP/IRE interaction stabilizes TfR1 mRNA and increases TfR1 production. Unlike TfR1, the expression of TfR2 is not expressed ubiquitously and is not influenced by intracellular iron levels [19], [26], [30]. The monoclonal antibodies used in the course of this study were those against TfR1.

In the present study, quantitative analysis of IF images of human specimens from the colon epithelium of Crohn's patients and normal specimens showed that TfR expression was higher at the membrane of colonocytes in IBD patients (**Figures 2** **and** **3**) and in colitis-induced rats (**Figure 4**). Extracting the mucosal proteins from the rat colons, followed by immunoblotting showed a lesser difference in TfR levels (1.4-fold, **Figure 5**). The observation that the relative expression of TfR in the mucosa of the inflamed colon of the rat was lower than that of human specimens compared with normal mucosa, suggests that the manner by which inflammation is induced involves membrane trafficking of TfR1, while the total intracellular pool of the receptor is less affected. Interestingly, a 4-fold enrichment of cell surface TfR levels was reported in malignant breast cells when compared to non-neoplastic breast cells [19]. Thus, under harsh inflammatory conditions TfR may serve as a therapeutic target similar to its use in cancer. Because TfR is upregulated in proliferating cells it is not surprising that both T and B lymphocytes express high levels of TfR in DSS-induced mice [31]. Nonetheless, the regulation of TfR1 expression in macrophages is more complicated. While exposure of macrophages to LPS and IFNγ causes down regulation of surface TfR1 [32], downstream signals, independent of IRP, enhance TfR transcription and promotes its expression [16], [33]. Only a small number of studies addressed TfR expression in gut mucosal cells in IBD. In a series of studies, Pallone et al showed that HLA-DR, 4F2 antigen and TfR are upregulated in IBD. Sixteen of 24 mucosal specimens from colitis patients (compared with none of 7 normal biopsies) were stained positively to TfR [15], [34], [35], [36], [37]. However, because of technical limitations related to the year in which these studies were conducted, the quality of the images and lack of quantification precluded a clear conclusion to whether TfR expression is elevated on colonocytes themselves in colitis. Here, two decades later, with improved imaging technology, we clearly show that TfR is expressed on both the apical and the basolateral membranes of the colonocytes in a manner directly correlated with the severity of the disease (**Figure 2**). While in the non-inflamed controls TfR was confined to the basolateral membrane, in the biopsies taken from the colonic mucosa of Crohn's patients TfR was enriched also at the apical surface (**Figure 3**). This is in contradiction to previous reports dealing with Madin-Darby canine kidney (MDCK) cells and Caco-2 cells, in which TfR expression was 40-fold higher in the basolateral than in the apical membranes. The sorting signal of TfR to the basolateral surface was ascribed to tyrosine motifs in its cytoplasmic tail [38], [39], [40]. A possible explanation to the findings in our models is the injury caused by the inflammation which may perturb the integrity of the tight junctions separating the basolateral from the apical membranes, allowing basolateral TfR to diffuse apically across the tight junctions. Indeed, proinflammatory mediators, such as IL-13, IFNγ and TNFα have been shown to disrupt and dysfunction tight junctions [41]. It remains to determine whether, in IBD, other typical basolateral proteins relocalize towards the apical side of the cells, indicating a general fault in cell polarity.

Another concern we had was that the increased level of TfR was perhaps secondary to the development of anemia and not due to direct effect on TfR regulation. Of the 9 specimens from Crohn's patients, at least four had overt clinical signs of anemia. Nevertheless, no signs of anemia were observed in colonic mucosa specimens from the colitis-induced rats, as indicated by various hematological parameters, despite clear elevation in TfR expression (**Figure S1 and S2**). To further explore the inflammation-mediated TfR regulation, we tested whether proinflammatory cytokines affect TfR levels *in vitro*. TNFα is most likely the most important proinflammatory cytokine in the pathogenesis of Crohn's disease as evident by the efficacy of anti-TNFα therapy in the clinics [1], [10], [42]. Furthermore, it has been reported that the exposure of Caco-2 cells to a mixture of TNFα, IL1β and IL-6 increased transferrin-iron uptake by 70%, inferring that proinflammatory cytokines are capable of modulating iron homeostasis in intestinal epithelial cells [28]. For this reason we analyzed TfR levels in Caco-2 cells subjected to cytokines treatment. We found that TNFα was capable of increasing TfR levels *in vitro* in a mechanism which does not involve hyper proliferation. In our hands IL-6 and IL-1β did not affect TfR (**Figures 5** **and** **7**). It would be interesting to test whether this effect of TNFα can be subverted by anti-inflammatory cytokines. Regardless, our data demonstrate a direct role of inflammation in the regulation of TfR expression level.

Does apical TfR play a functional role in retrieving iron? One third of IBD patients suffer from recurrent anemia due to iron deficiency [30], [43]. Physiologically, TfR is not involved in iron absorption from the gut. However, because transferrin is accumulated at the apical side of colonocytes in IBD, the simultaneous enhancement in apical TfR levels could lead to reabsorbtion of the leaked transferrin. If true, apical TfR could serve as a targeting molecule in the design of specific drug delivery vehicles for the treatment of IBD, owing to its location at the inflamed sites and the possibility that it may trigger endocytosis upon ligation. TfR targeting has been extensively studied in the context of cancer therapy [26], [27]. To this end TfR was targeted by virtue of its ligand Tf, as well as by specific monoclonal antibodies or single chain antibody fragments directed to its extracellular domain [27], [44], [45]. To explore the potential of TfR as a means to direct drug vehicles to the inflamed mucosa we prepared negatively charged liposomes and conjugated them with anti-TfR antibodies. Anti-TfR was favored over Tf due to three reasons: first, endogenous Tf which accumulate in the inflamed mucosa [14] may compete with Tf-bound liposomes for TfR binding. In contrast, anti- TfR does not bind to the Tf binding site and therefore less susceptible to direct competition. Secondly, the pH of inflamed tissue becomes acidic. At pH lower than 5, Tf acquires a positive charge which may form electrostatic interactions with the negatively charged liposomes. The antibody is most likely more resilient to changes in pH values. Lastly, at acidic pH, typical to the colon lumen in IBD, Tf forms a stable complex with TfR, which enable the release of iron and recycling Tf back to the cell surface [26]. The acidic ambience in the inflamed colon could potentially cause Tf to release the ferric ions and lose affinity to the receptor.

Although not directly addressed in this study, we decided to generate immunoliposomes as our model for drug delivery vehicle owing to their capability to entrap a variety of drugs for the topical treatment of IBD. In addition, if made at the right size, they could adhere to the cellular membranes and deliver the drug payload by endocytosis [46]. Since the immunoliposomes are aimed at targeting mucosal tissues, the mucus barrier should not be underestimated. To overcome this, PEG was used as a spacer for the conjugation of the anti-TfR [47]. It has been reported that PEG effectively minimizes adhesive interactions between mucin mesh fibers and particles' surface [48], [49], [50]. Preparation of the liposomes at acidic conditions, which should theoretically increase their stability, did not improve the conjugation yield (**Figure** 1).

Upon incubation with Caco-2 and IEC-6 cells, the immunoliposomes were internalized (**Figures 8** **and** **9**), indicating that the liposomes were recognized by membrane TfR. Moreover, anti-TfR facilitated the apical uptake to the rat inflamed mucosa by more than 4-fold compared to their uptake by healthy mucosa (**Figures 10**). TfR expression may rise globally due to development of anemia or locally in healthy mucosal areas adjacent to the inflamed regions, owing to leakage of proinflammatory cytokines from the site of active inflammation to neighboring cells. If occurs, it may allow preferential delivery of drugs to areas prone to exert inflammation as the disease progresses. Obviously, because of these reasons and because basal levels of TfR are expressed in the healthy duodenum and proximal small intestine [27], liposomes equipped with anti-TfR antibody are not suitable for drug delivery via the oral route as is. However, once delivered to the distal organs of the alimentary canal by either colonic delivery systems or by enema, their homing to the sites of injury will be facilitated. This assumption requires further verification. Inasmuch, we do not know at this point whether at an in vivo setting anti-TfR immunoliposomes will penetrate the enterocytes by transcytosis, as suggested by Widera et al [26], or remain confined to the apical site.

Overall, our findings delineate, for the first time that mucosal TfR is directly regulated by proinflammatory cytokines and that this regulation could be exploited for colon-specific delivery of drugs, for the local treatment of IBD.

## Supporting Information

Scheme S1 **Preparation of the αTfR immunoliposomes.** The antibody solution was added to an aqueous dispersion of negatively charged liposomes, bearing NHS-PEG-DSPE handle and tagged with carboxyfluorescein (CF), right after their preparation. Conjugation was accomplished by an overnight agitation at room temperature, after which the reaction was stopped by the addition of a TRIS buffer.
(TIF)

Figure S1 **Characterization of DNBS induced colitis.** Scoring of the colon of saline treated or DNBS induced colitis. The induced rats showed high score (total score of 4) as measured by weight lost, diarrhea, colon weight, hyperemia, edema, ulcerated areas and shorten of the colon length.
(TIF)

Figure S2 **DNBS induced colitis do not develop anemia.** Blood count: blood count for healthy and inflamed rats: (a) white blood cells count. (b) Red blood cells count. (c) Hemoglobin (d) Hematocrit. (e) Mean corpuscular volume. (f) Mean Cell Hemoglobin. (g) Platelets. The tests were performed in veterinary division of American medical Laboratories, AML, ISREAL, LTD. Healthy (N = 3), Inflammation (N = 4).
(TIF)
