# Supplementary figures and images for: Enhanced Transferrin Receptor Expression by Proinflammatory Cytokines in Enterocytes as a Means for Local Delivery of Drugs to Inflamed Gut Mucosa
Source: PLoS One. 2011 Sep 6;6(9):e24202. doi: 10.1371/journal.pone.0024202 (PMC3167832; doi:10.1371/journal.pone.0024202)

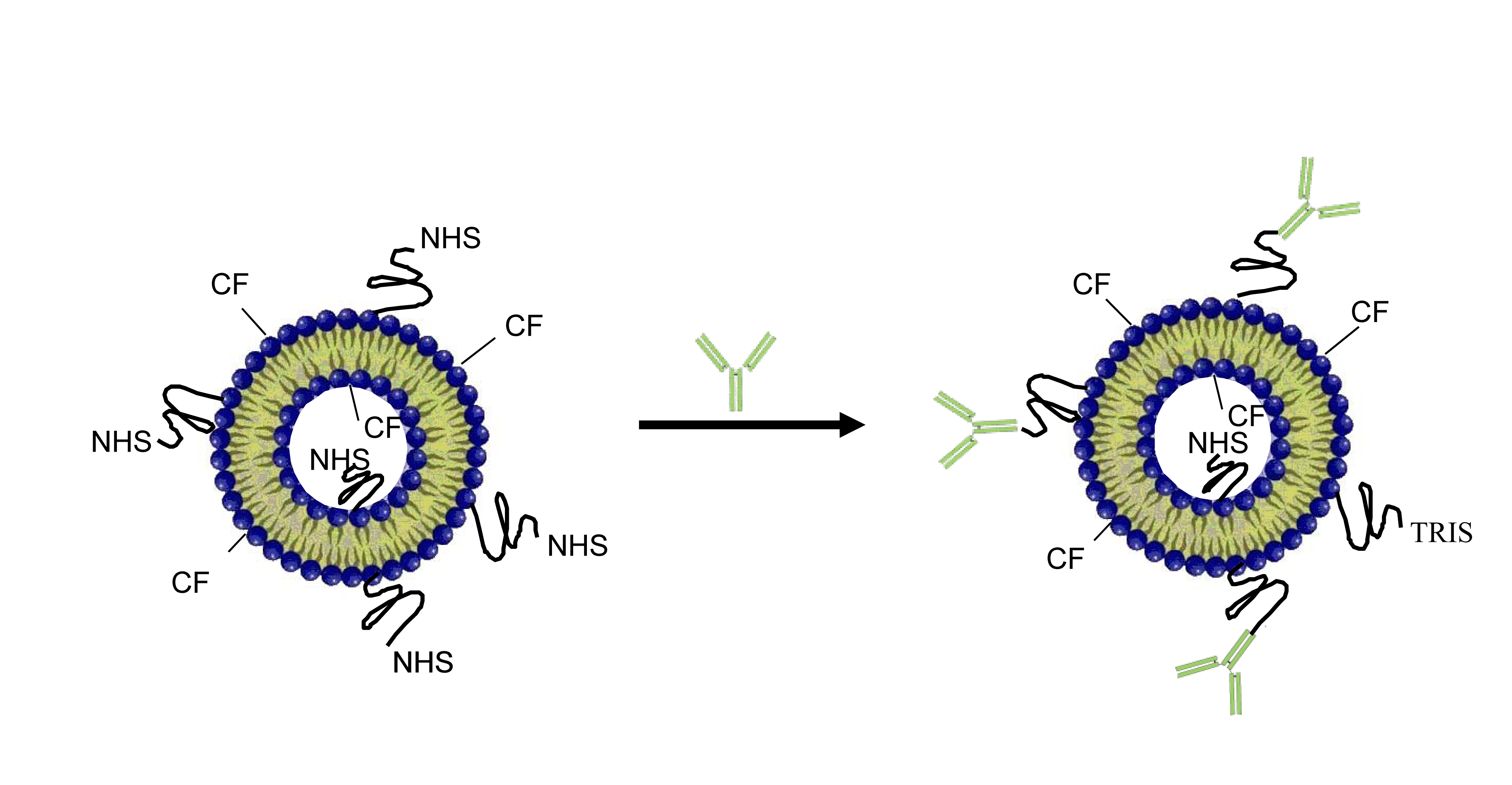

Supplement: Scheme S1 — Preparation of the αTfR immunoliposomes. The antibody solution was added to an aqueous dispersion of negatively charged liposomes, bearing NHS-PEG-DSPE handle and tagged with carboxyfluorescein (CF), right after their preparation. Conjugation was accomplished by an overnight agitation at room temperature, after which the reaction was stopped by the addition of a TRIS buffer. (TIF) [file pone.0024202.s001.tif]

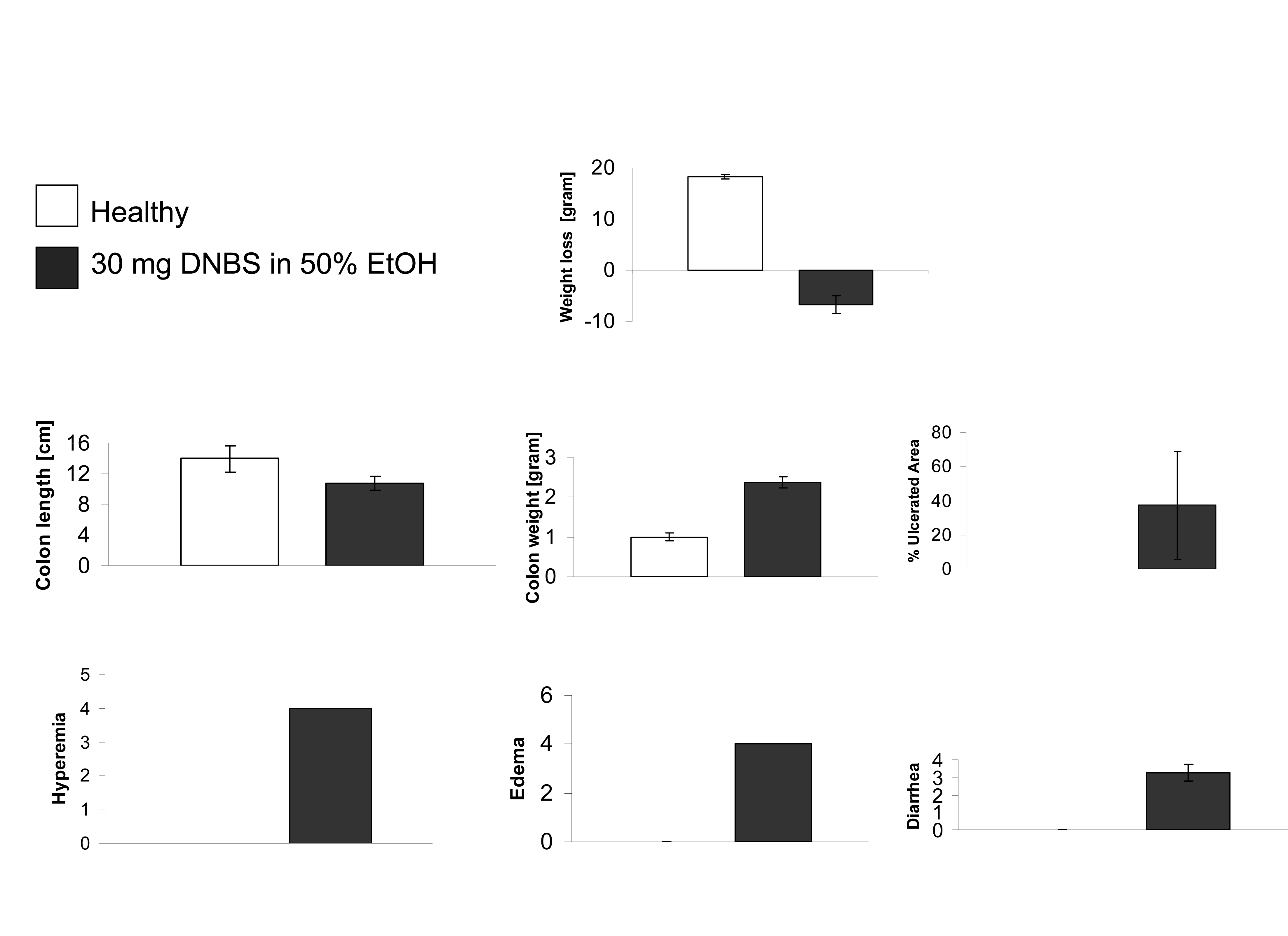

Supplement: Figure S1 — Characterization of DNBS induced colitis. Scoring of the colon of saline treated or DNBS induced colitis. The induced rats showed high score (total score of 4) as measured by weight lost, diarrhea, colon weight, hyperemia, edema, ulcerated areas and shorten of the colon length. (TIF) [file pone.0024202.s002.tif]

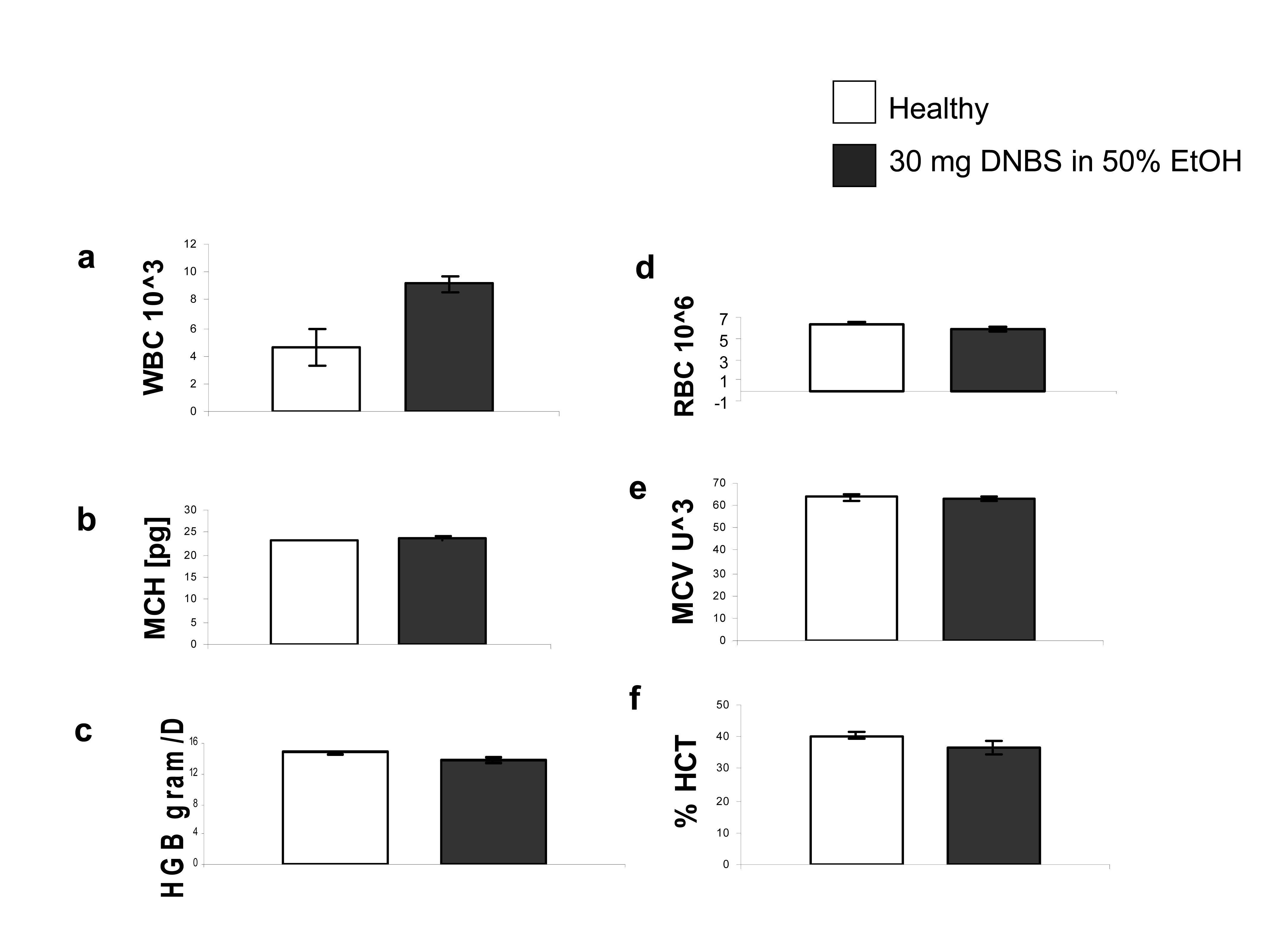

Supplement: Figure S2 — DNBS induced colitis do not develop anemia. Blood count: blood count for healthy and inflamed rats: (a) white blood cells count. (b) Red blood cells count. (c) Hemoglobin (d) Hematocrit. (e) Mean corpuscular volume. (f) Mean Cell Hemoglobin. (g) Platelets. The tests were performed in veterinary division of American medical Laboratories, AML, ISREAL, LTD. Healthy (N = 3), Inflammation (N = 4). (TIF) [file pone.0024202.s003.tif]
